# Risk and outcomes of breakthrough COVID‐19 infections in vaccinated immunocompromised patients: A meta‐analysis

**DOI:** 10.1002/mco2.307

**Published:** 2023-06-09

**Authors:** Guangtong Deng, Qian Zhou, Yu Meng, Huiyan Sun, Songtao Du, Yihuang Liu, Furong Zeng

**Affiliations:** ^1^ Department of Dermatology Hunan Engineering Research Center of Skin Health and Disease Hunan Key Laboratory of Skin Cancer and Psoriasis Xiangya Hospital Central South University Changsha Hunan China; ^2^ National Clinical Research Center for Geriatric Disorders Xiangya Hospital Central South University Changsha Hunan China; ^3^ Department of Colorectal Surgical Oncology the Tumor Hospital of Harbin Medical University Harbin Heilongjiang China; ^4^ Department of Oncology Xiangya Hospital Central South University Changsha Hunan China

Dear Editor,

Breakthrough infection, which is defined as reinfection of coronavirus disease 2019 (COVID‐19) after vaccination with a typical 14‐day lag period, has been recorded.^1^ However, concerns have been raised regarding the potential risk and outcomes of breakthrough COVID‐19 infections between immunocompromised and immunocompetent patients.^2^, ^3^ To fill in the gaps, we performed the meta‐analysis with two key questions explored: are immunocompromised patients related to an increased risk of breakthrough COVID‐19 infections, hospitalization, and mortality; and are different subtypes of immunocompromised patients associated with different risk and outcomes of breakthrough COVID‐19 infections?

This meta‐analysis was based on the Preferred Reporting Items for Systematic Reviews and Meta‐analyses guidelines. The registration number was CRD42022360524. Databases including Pubmed, Embase, and Cochrane Library were searched from inception to May 19, 2023. No restrictions were placed on publication languages and study types (Table S1). The inclusion criteria were as follows: (1) cohort or case–control studies with adult participants; (2) adjusted risk ratios (RRs), odd ratios (ORs), or hazard ratios and their 95% confidence intervals (CI) for the risk or outcomes (including hospitalization and mortality) of breakthrough COVID‐19 infections between vaccinated immunocompromised and immunocompetent patients. Immunocompromised patients were defined as patients with cancer, active immune mediated inflammatory disorders (IMID) except asthma, solid organ transplant (SOT), or human immunodeficiency virus (HIV)/acquired immune deficiency syndrome (AIDs).^3^ Immunocompetent patients were people who were not immunocompromised. We excluded case reports, case series, and studies for which data were not available from the corresponding author. We graded the quality of all included studies as high quality (at least seven stars), moderate quality (four to six stars), or low quality (less than four stars) using the Newcastle‐Ottawa Scale (NOS).

The primary outcomes were the risk and outcomes of breakthrough COVID‐19 infections between vaccinated immunocompromised and immunocompetent patients. The secondary outcomes were how the risk and outcomes varied among different subtypes of immunocompromised diseases. RRs with 95% CI were combined by the inverse variance approach. We preferentially use DerSimonian and Laird random‐effect model rather than the fixed‐effect model as the primary method to pool results across studies due to underlying clinical heterogeneity.^4^ Subgroup analyses, sensitivity analyses, and publication bias were used to further assess the consistency of the pooled results. All the analyses were conducted and visualized using R software (3.6.3) and STATA 12SE. *p* < 0.05 was regarded statistically significant.

Our initial search returned 1246 studies through database mining (Figure 1A). After screening, 25 studies including 28 cohorts were finally enrolled in the meta‐analysis. The main characteristics of the enrolled studies were shown in Table S2. Notably, 13 cohorts had adequate adjustment, while 15 cohorts did not adjust for any confounders (Table S3). The quality of the included studies was evaluated according to the NOS tool, which demonstrated that 26 cohorts were graded as good quality and two as moderate quality (Table S4).

**FIGURE 1:**
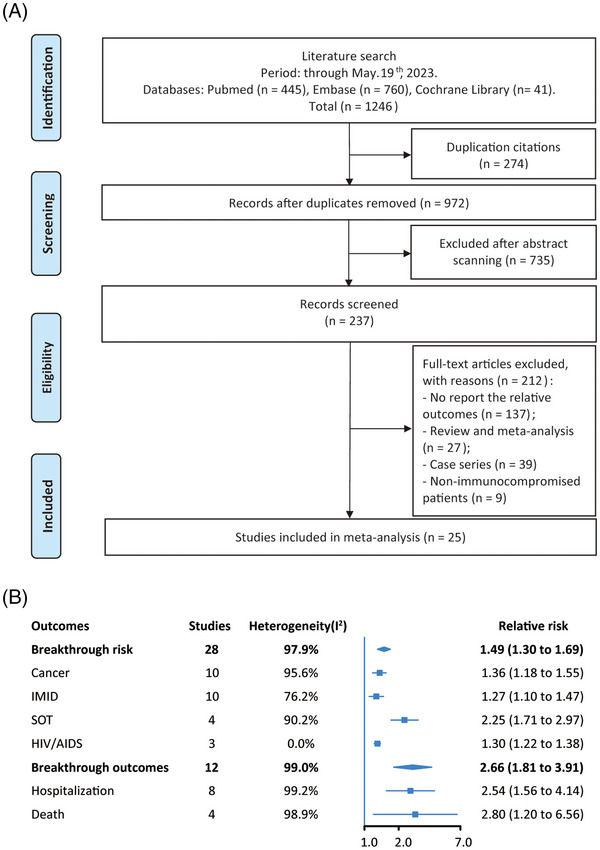
Risk ratios for breakthrough COVID‐19 infections and severe outcomes in vaccinated immunocompromised patients. (A) Flowcharts illustrating the article selection process. (B) Risk ratios for breakthrough COVID‐19 infections and severe outcomes in vaccinated immunocompromised patients compared with immunocompetent controls. IMID, immune‐mediated inflammatory disorders; SOT, solid organ transplant; HIV, human immunodeficiency virus; AIDs, acquired immune deficiency syndrome.

As shown in Figure 1B, vaccinated immunocompromised patients were associated with an increased risk of breakthrough COVID‐19 infection (RR 1.49, 95%CI, 1.30–1.69). Among the immunocompromised diseases, relative risks were highest for patients with SOT (RR 2.25, 1.71–2.97), followed by those with cancer (RR 1.36, 1.18–1.55), IMID (RR 1.27, 1.10–1.47), and HIV/AIDS (RR 1.30, 1.22–1.38). We observed significant heterogeneity for the risk of all comparisons (all *I* ^2^ > 50%, *p* < 0.10) except for HIV/AIDs patients (*I* ^2^ = 0%, *p* = 0.53). Univariate meta‐regression suggested that COVID‐19 history (exp (*β*) 0.68, 0.49–0.94, *p* = 0.02) and countries (exp (*β*) 1.51, 1.08–2.10, *p* = 0.02) were significant sources of heterogeneity in vaccinated immunocompromised patients (Table S5). Besides, COVID‐19 history (exp (*β*) 0.63, 0.41–0.96, *p* = 0.04) and vaccine type (exp (*β*) 1.56, 1.02–2.37, *p* = 0.04) were also possible significant moderators of heterogeneity in patients with IMID. We did not detect the existence of publication bias in these studies via the funnel plot and Egger's test (*p* = 0.61) (Figure S2A). Sensitivity analyses confirmed consistent results when using fixed‐effect model (RR 1.35, 1.33–1.37) (Figure 1A), or only including the studies that present the outcomes as hazard ratios (HR 1.49, 1.20–1.84) (Figure S2B), or removing one study at a time from the analysis (Figures S2C and S3), confirming the stability of the results. All the results of subgroup analyses showed an increased risk of breakthrough COVID‐19 infection in vaccinated immunocompromised patients (RR range, 1.04–1.97), including those with cancer (RR range, 1.02–1.77) and IMID (RR range, 1.07–1.54) (Table S6).

Consistently, compared with immunocompetent controls, the risk of breakthrough COVID‐19 infection in solid cancers patients was significantly increased (RR 1.22, 1.11–1.33) (Figure S4). All the solid cancer subtypes were related to an increased risk of breakthrough COVID‐19 infection (RR range 1.04–1.36). We also found that patients with hematologic cancers were related to an increased risk of breakthrough COVID‐19 infection (RR 2.93, 2.11–3.87) (Figure S5). Significant RRs were observed for the risk of breakthrough COVID‐19 infection among all hematological cancer subtypes, including lymphoma (RR 2.39, 1.76–3.25), leukemia (RR 3.24, 1.83–5.71), and multiple myeloid (RR 3.41, 1.47–7.89). Moreover, as illustrated in Figure S6, vaccinated patients with systemic rheumatic diseases were related to an increased risk of breakthrough COVID‐19 infection (RR 1.23, 1.18–1.29) with minimal heterogeneity (*I* ^2^ = 0%, *p* > 0.10).

Furthermore, immunocompromised patients had significantly higher risk for severe outcomes (hospitalization and mortality), compared with immunocompetent control (RR 2.66, 1.81–3.91). Subgroup analysis showed that vaccinated immunocompromised patients were associated with an increased risk of hospitalization (RR 2.54, 1.56–4.14), and mortality (RR 2.80, 1.20–6.56) (Figures 1B and S7). We could not completely exclude possible publication bias due to a little asymmetry in the funnel plot (Figure S8A), while no publication bias was found using Egger's test (*p* = 0.82). However, the trim‐and‐fill analysis did not change the results. Sensitivity analyses further confirmed that the outcomes did not change when excluding one study at a time from the analysis (Figures S8B–D), or using fixed‐effect model (RR 3.12, 3.02–3.21) (Figure S7). Univariate meta‐regression did not find significant sources of heterogeneity for the outcomes (Table S7). Subgroup analyses showed immunocompromised patients were associated with an increased risk for severe outcomes (RR range, 2.17–3.69), without significant heterogeneity among all subgroup comparisons (all *p* > 0.05) (Table S8).

Undeniably, the study had some limitations. First, notable heterogeneity was observed in several comparisons, though sensitivity analyses, subgroup analyses and trim‐and‐fill analyses were performed to confirm the stability of the results. Second, there were currently more than nine different COVID‐19 vaccines in the global market, but nearly all the data included mRNA vaccines and few of them focus on other types of vaccines. Third, SARS‐CoV‐2 has evolved multiple variants.^5^ Due to data unavailability, whether conclusions were consistent in different variants still needs to be further investigated. Finally, the included studies were mainly from USA and Israel, and we should further validate the conclusions among developing countries.

In aggregate, vaccinated immunocompromised patients were related to an increased risk of breakthrough COVID‐19 infections, hospitalization, and mortality compared with vaccinated immunocompetent patients.

## AUTHOR CONTRIBUTIONS

Furong Zeng and Guangtong Deng contributed to the design of the study; Guangtong Deng and Qian Zhou performed data acquisition; Guangtong Deng and Furong Zeng performed data analysis and interpretation; Guangtong Deng and Qian Zhou drafted the manuscript; Qian Zhou, Yu Meng, Huiyan Sun, Songtao Du and Yihuang Liu performed manuscript revision. All authors have read and approved the final manuscript.

## CONFLICT OF INTEREST STATEMENT

All authors report no conflict interest relevant to this article.

## FUNDING INFORMATION

This work was supported by the National Natural Science Foundation of China (Grant Nos. 82103183 to F. Z., 82102803, 82272849 to G. D.), Natural Science Foundation of Hunan Province (Grant Nos. 2022JJ40767 to F. Z., 2021JJ40976 to G. D.) and Natural Science Fund for Outstanding Youths in Hunan Province (2023JJ20093 to G. D.).

## ETHICS STATEMENT

No ethical approval was needed.

## Supporting information

Supporting Information

## Data Availability

The datasets generated during and/or analyzed during the current study are available from the corresponding author on reasonable request.
