# Supplementary material for: Risk and outcomes of breakthrough COVID‐19 infections in vaccinated immunocompromised patients: A meta‐analysis
Source: MedComm (2020). 2023 Jun 9;4(3):e307. doi: 10.1002/mco2.307 (PMC10256991; doi:10.1002/mco2.307)
Supplement: Supplementary file 1 — Supporting Information [file MCO2-4-e307-s001.docx]

Supplemental Materials

**Risk and outcomes of breakthrough COVID-19 infections in vaccinated immunocompromised patients: a meta-analysis**

Guangtong Deng^1-2#^, Qian Zhou^1-2#^, Yu Meng^1-2^, Huiyan Sun^1-2^, Songtao Du^3^, Yihuang Liu^1-2^, Furong Zeng^4*^

^1^ Department of Dermatology, Hunan Engineering Research Center of Skin Health and Disease, Hunan Key Laboratory of Skin Cancer and Psoriasis, Xiangya Hospital, Central South University, Changsha, Hunan 410008, China

^2^ National Clinical Research Center for Geriatric Disorders, Xiangya Hospital, Central South University, Changsha, Hunan 410008, China

^3^ Department of Colorectal Surgical Oncology, the Tumor Hospital of Harbin Medical University, Harbin, Heilongjiang 150000, China

^4^ Department of Oncology, Xiangya Hospital, Central South University, Changsha, Hunan 410008, China.

^#^Guangtong Deng and Qian Zhou contributed equally to this work.

*Correspondence Furong Zeng

**This file includes:**

Figures S1 to S8

Tables S1 to S8

References

**Supplemental Figures**


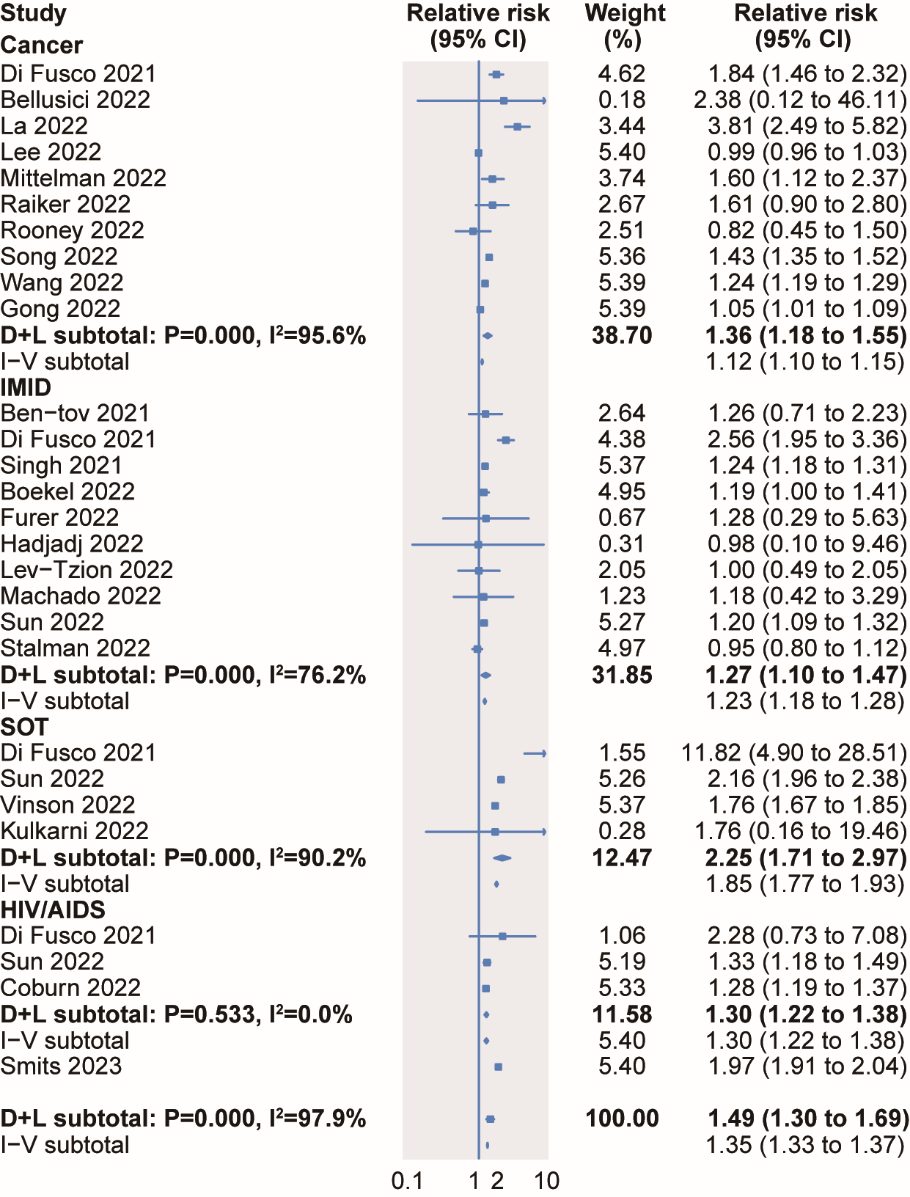


**Figure S1.** Risk ratios for breakthrough COVID-19 infections in vaccinated immunocompromised patients compared with immunocompetent controls. IMID: immune-mediated inflammatory disorders; SOT: solid organ transplant; HIV: human immunodeficiency virus; AIDs: acquired immune deficiency syndrome.


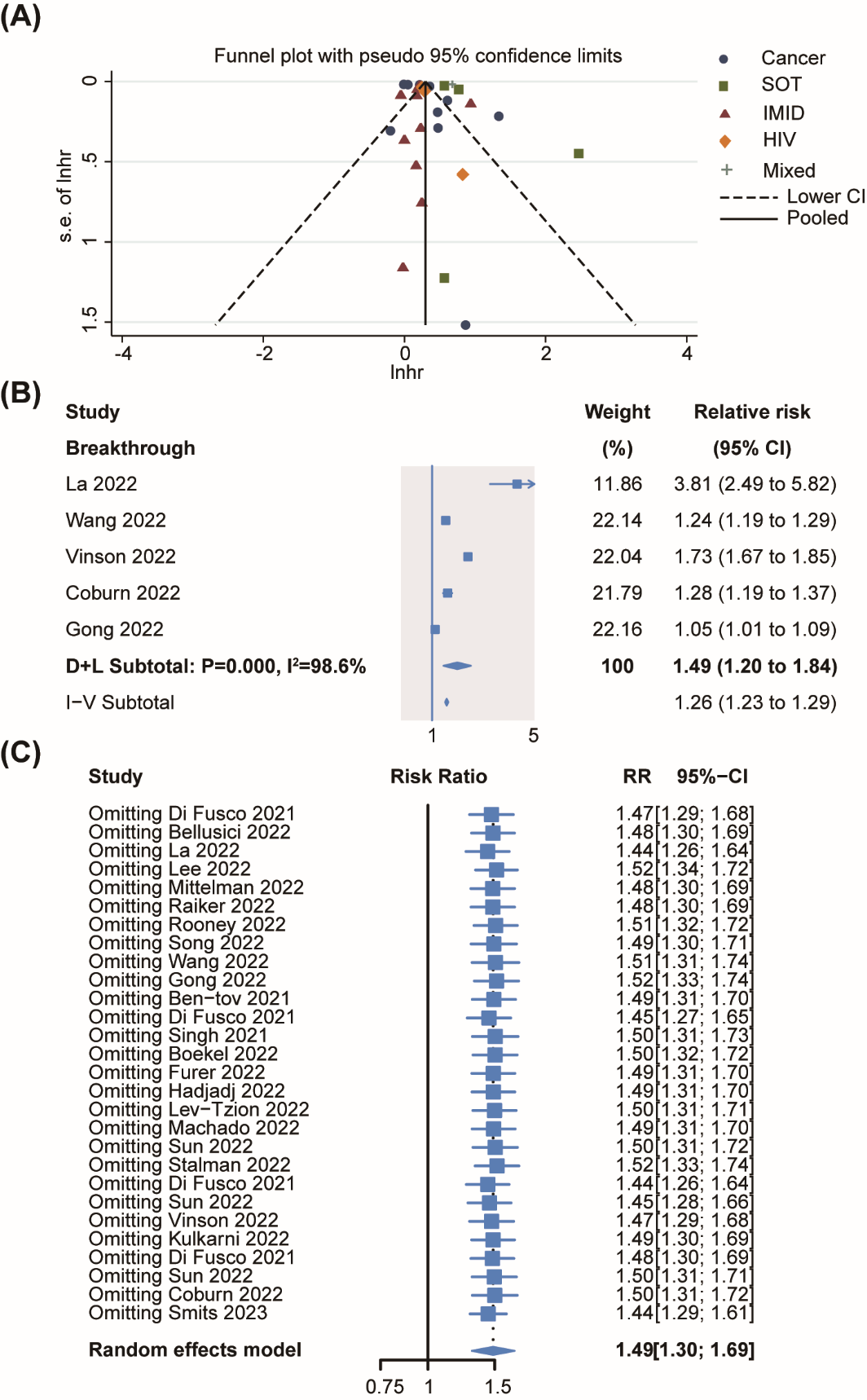


**Figure S2**. The funnel plot (A), the subgroup analysis only including the studies that present the outcomes as hazard ratios (B), and the sensitivity analysis using “leaving-one-out” per time approach (C) for breakthrough COVID-19 infections in vaccinated immunocompromised patients compared with immunocompetent controls.


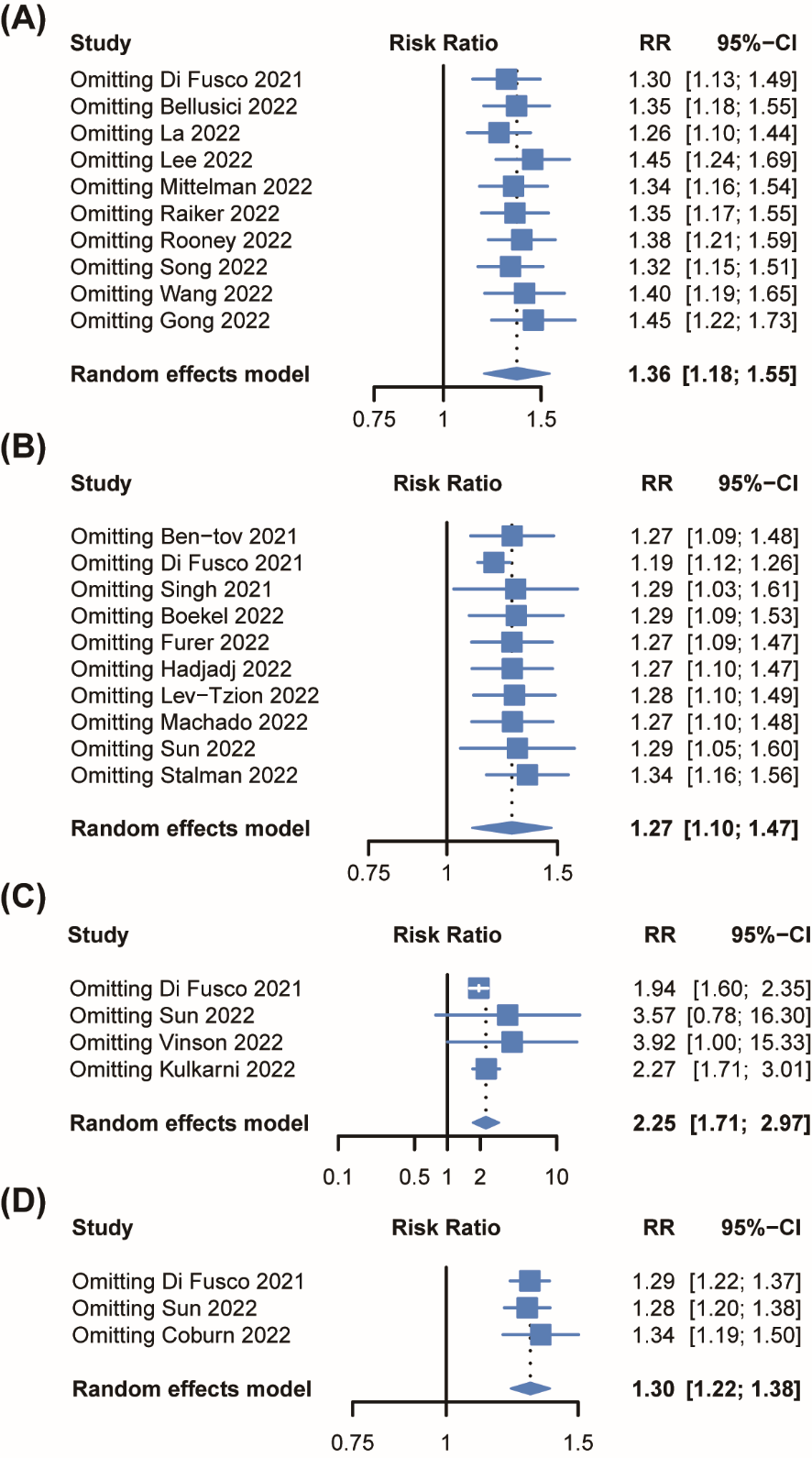


**Figure S3**. The sensitivity analysis using “leaving-one-out” per time approach for breakthrough COVID-19 infections in vaccinated immunocompromised patients with cancer (A), immune-mediated inflammatory disorders (B), organ transplant (C), or HIV/AIDS (D) compared with immunocompetent controls.


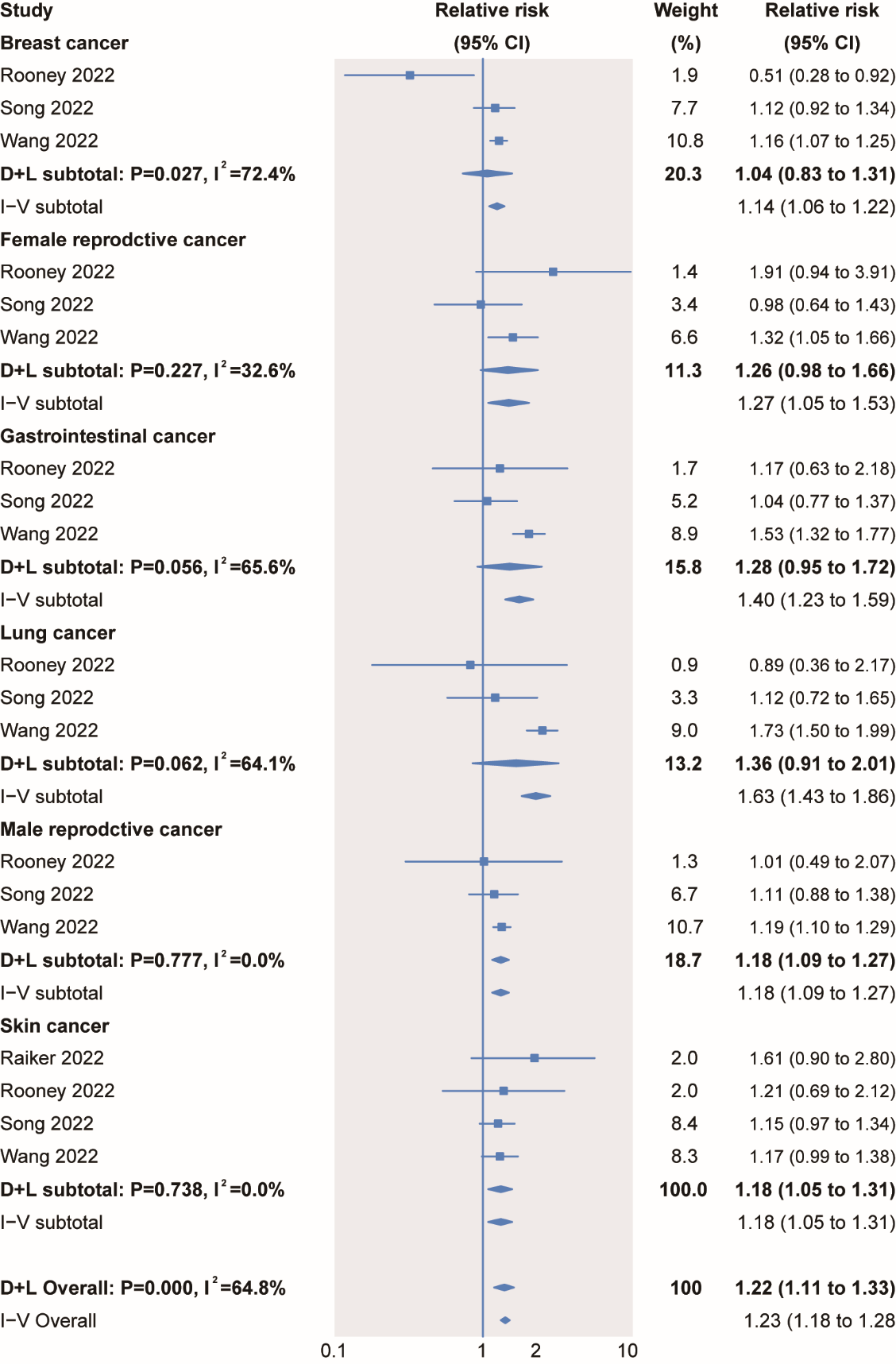


**Figure S4**. Risk ratios for breakthrough COVID-19 infections in vaccinated solid cancer patients compared with immunocompetent controls.


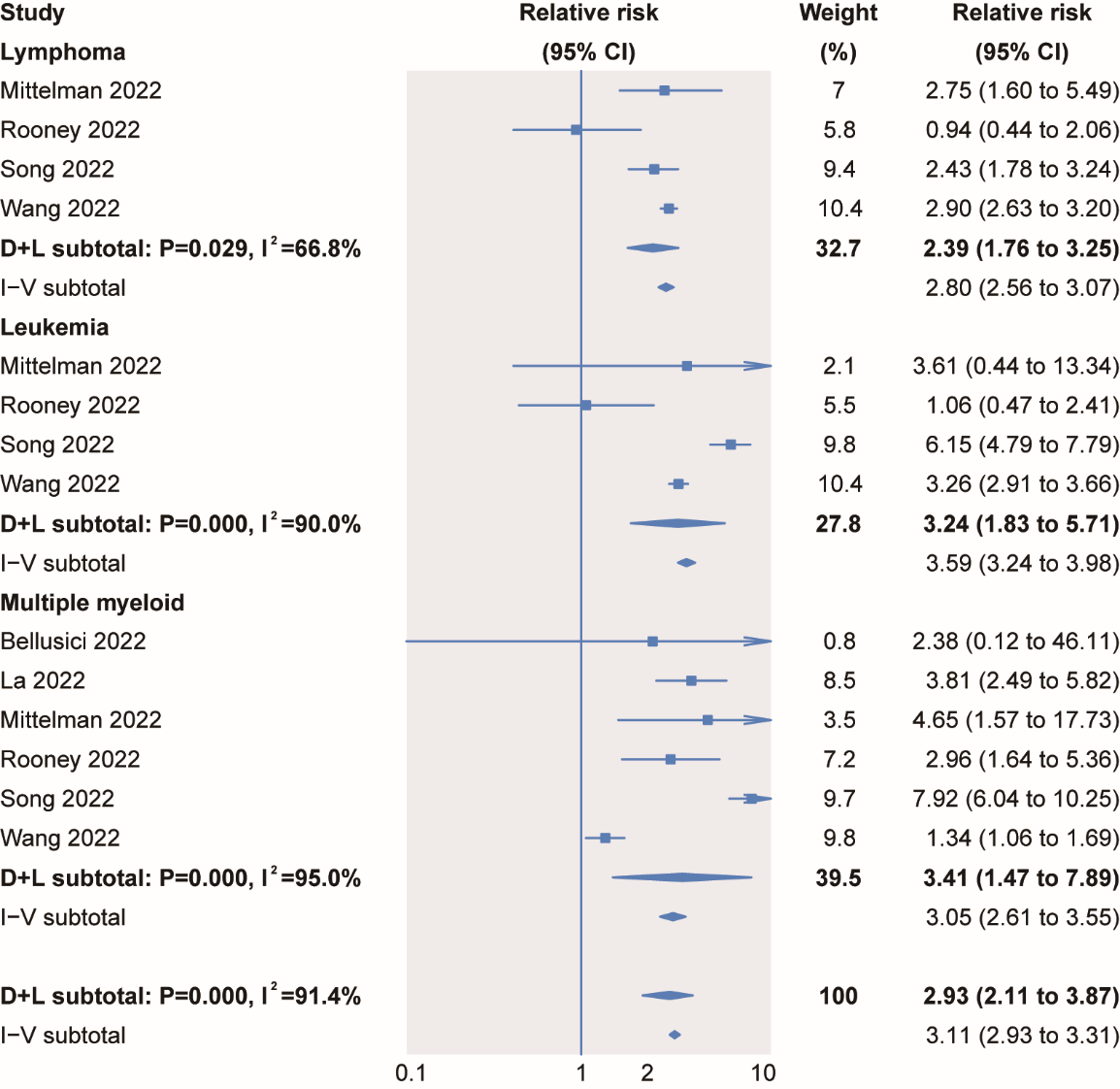


**Figure S5**. Risk ratios for breakthrough COVID-19 infections in vaccinated hematological cancer patients compared with immunocompetent controls.


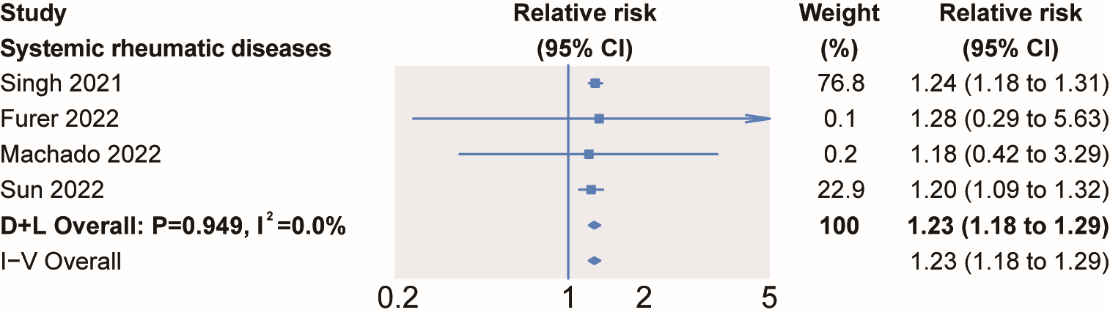


**Figure S6**. Risk ratios for breakthrough COVID-19 infections in patients with systemic rheumatic diseases compared with immunocompetent controls.


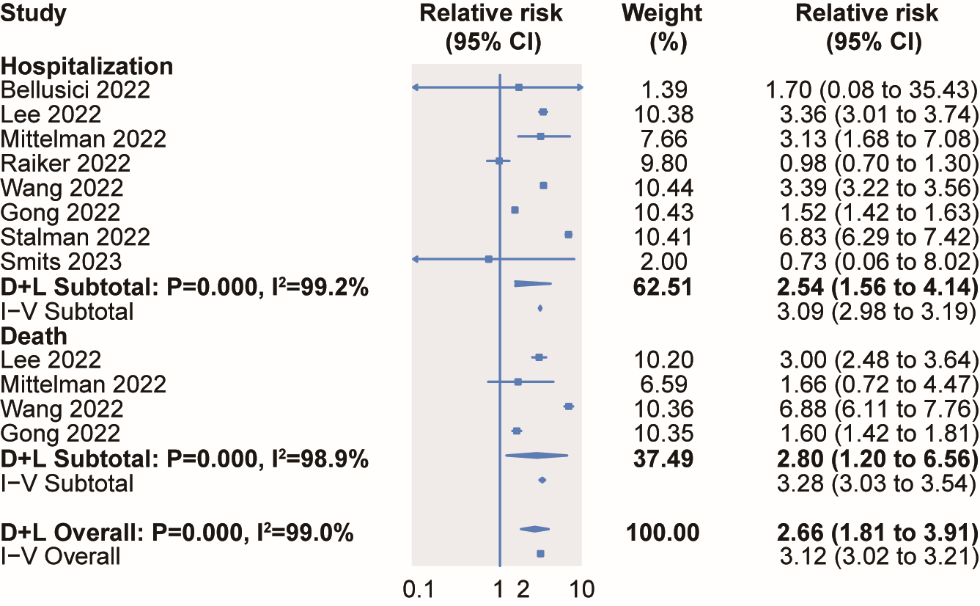


**Figure S7**. Risk ratios for severe outcomes (hospitalization and mortality) in vaccinated immunocompromised patients compared with immunocompetent controls.


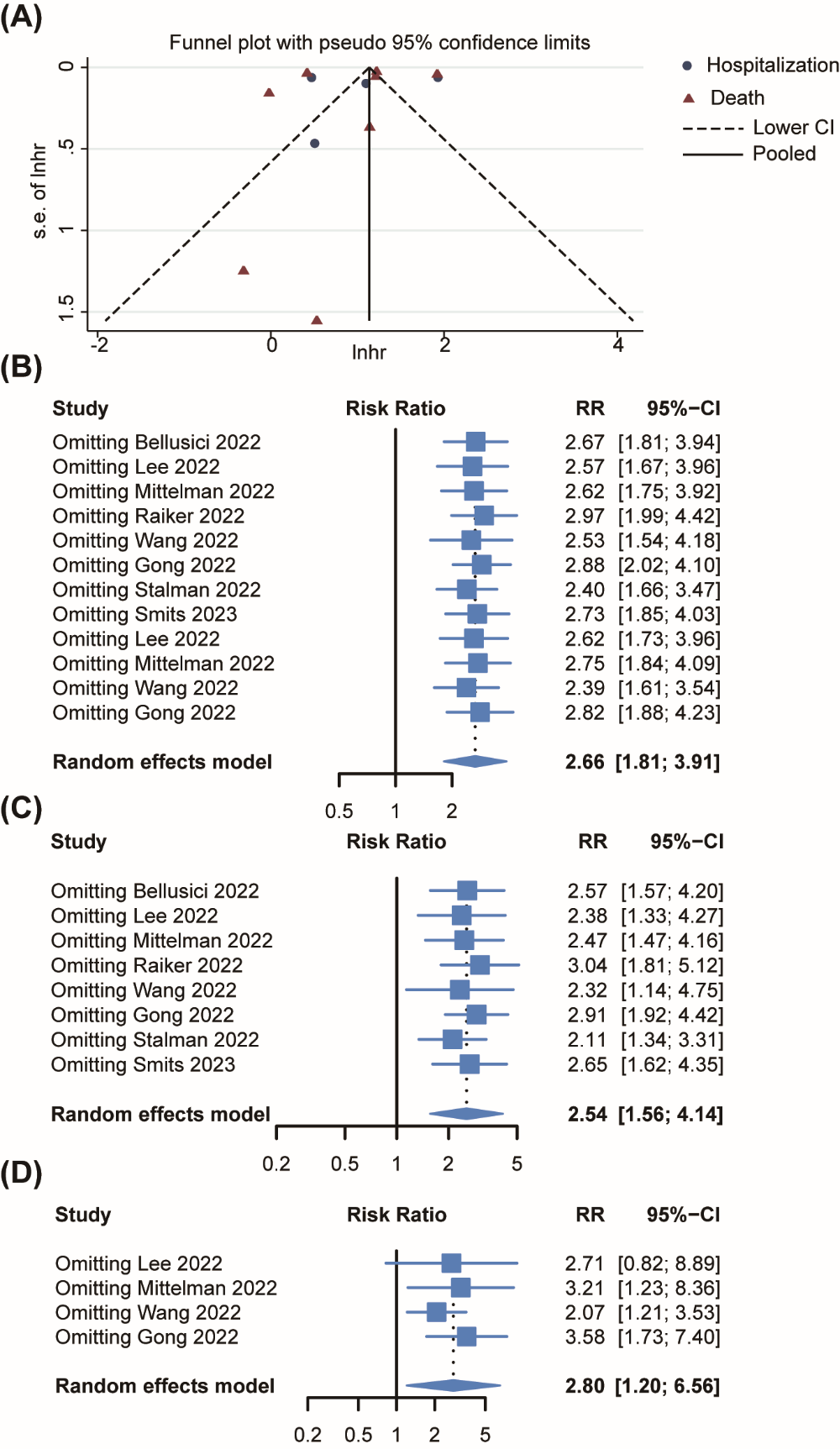


**Figure S8**. The funnel plot (A), and the sensitivity analysis using “leaving-one-out” per time approach for the severe outcomes (B), hospitalization (C), and mortality (D) of breakthrough COVID-19 infections in vaccinated immunocompromised patients compared with immunocompetent controls.

**Table S1. The detailed search strategy.**

| Databases | Search strategies | Articles’ number |
| --- | --- | --- |
| Pubmed | #1 corona[ti] OR covid*[ti] OR sars[ti] OR severe acute respiratory syndrome[ti] OR ncov*[ti] OR "severe acute respiratory syndrome coronavirus 2" [Supplementary Concept] OR "COVID-19" [Supplementary Concept] OR (wuhan[tiab] AND coronavirus[tiab]) OR (wuhan[tiab] AND pneumonia virus[tiab]) OR COVID19[tiab] OR COVID-19[tiab] OR coronavirus 2019[tiab] OR SARS-CoV-2[tiab] OR SARS2[tiab] OR SARS-2[tiab] OR "severe acute respiratory syndrome 2"[tiab] OR 2019-nCoV[tiab] OR (novel coronavirus[tiab] AND 2019[tiab]) NOT (animals[mesh] NOT humans[mesh])  #2 (“Vaccines”[MeSH] OR “vaccination”[MeSH] OR vaccine[All Fields] OR vaccination[All Fields] OR vaccin*[All Fields])  #3 “Breakthrough” OR “Breakthrough infection” OR “vaccine-breakthrough” OR “Breakthrough case” OR “vaccinated infection”  #4 (“Neoplasms”[MeSH] OR “cancer” OR “malignancy” OR “malign*” OR “immunocompromise”[All Fields] OR “immunocompromised”[All Fields] OR“immunodeficiency”[All Fields] OR “immunodeficient”[All Fields] OR “immunodef*”[All Fields] OR “immunocompr*”[All Fields] OR “chemotherapy”[All Fields] OR “chemo*”[All Fields] OR “immunosuppressed”[All Fields] OR “immunosuppression”[All Fields] OR “immunosupp*”[All Fields] OR “rheumatology”[All Fields] OR “rheumatic”[All Fields] OR “rheum*”[All Fields] OR “autoimmune”[All Fields] OR “autoimmunity”[All Fields] OR “transplant”[All Fields] OR “solid organ”[All Fields] OR steroids[MeSH] OR antineoplastic agents[MeSH] OR chemotherapy[MeSH] OR "Cytotoxicity, Immunologic"[Mesh] OR antirheumatic agents[MeSH] OR immunosuppressive agents[MeSH] or steroid* or corticosteroid* or (antineoplastic* AND agent*) OR chemotherap* or cytotoxic*)  #5 #1 AND #2 AND #3 AND #4 | 445 |
| Embase | #1 covid19 OR 'covid 19' OR 'sars cov 2' OR 'sars cov2' OR 'severe acute respiratory syndrome coronavirus 2' OR '2019 ncov' OR 2019ncov OR coronavirus  #2 'coronavirus infections'/exp  #3 'coronavirinae'/exp  #4 #1 OR #2 OR #3  #5 'vaccination'/exp OR vaccine OR vaccination OR vaccin*  #6 ‘Breakthrough’ OR ‘Breakthrough infection’ OR ‘vaccine-breakthrough’ OR ‘Breakthrough case’ OR ‘Vaccinated infection’  #7 ('cancer' OR 'malignancy' OR 'immunocom*' OR 'immunodef*' OR 'immunosupp*' OR 'immunomod*' OR 'immunocompromised patient'/exp OR 'immune deficiency'/exp OR 'malignant neoplasm'/exp OR 'chemotherapy'/exp OR 'autoimmune disease'/exp OR 'steroid*' OR 'transplant'/exp OR 'solid organ' OR 'immunosuppressive agent'/exp OR 'rheumatic disease'/exp OR immunosuppressed OR rheumatic OR rheumatoid OR autoimmune OR 'autoimmunity'/exp)  #8 #4 AND #5 AND #6 AND #7 | 760 |
| Cochrane Library | #1 MeSH descriptor: [COVID-19] explode all trees  #2 covid-19 or covid19 or covid-19 or sars-cov-2 or sars-cov2 or ‘severe acute respiratory syndrome coronavirus 2’or 2019ncov or coronavirus  #3 MeSH descriptor: [Coronaviridae] explode all trees  #4 #1 OR #2 OR #3  #5 ‘Breakthrough’ OR ‘Breakthrough infection’ OR ‘vaccine-breakthrough’ OR ‘Breakthrough case’ OR ‘Vaccinated infection’  #6 "vaccine" OR "vaccination" OR vaccination OR vaccin*  #7 "cancer" OR "malignancy" OR "malign*" OR "immunocompromise" OR "immunocompromised" OR "immunodeficiency" OR "immunodeficient" OR "immunodef*" OR "immunocompr*" OR "chemotherapy" OR "chemo*" OR "immunosuppressed" OR "immunosuppression" OR "immunosupp*" OR "rheumatology" OR "rheumatic" OR "rheum*" OR "autoimmune" OR "autoimmunity" OR "transplant" OR "solid organ" OR steroids OR antineoplastic agents OR chemotherapy OR cytotoxicity OR immunologic OR antirheumatic agents OR immunosuppressive agents or steroid* or corticosteroid* or (antineoplastic* AND agent*) OR (chemotherap* or cytotoxic*)  #8 #4 AND #5 AND #6 AND #7 | 41 |
| Duplications |  | -274 |
| Total after duplications |  | 972 |

**Table S2. Basic characteristics of included studies.**

| **Studies (years)** | **Country** | **Study type** | **Vaccine** | **Age** | **Gender (male. %)** | **COVID-19 history** | **N, patients of interest** | **N, comparison** | **Breakthrough (days)** | **Vaccine doses** |  |
| --- | --- | --- | --- | --- | --- | --- | --- | --- | --- | --- | --- |
| Smits (2023)  [1] | USA | RCS | BNT162b2, mRNA-1273 | Patients: NA, Control: NA | Patients: 38.0, Control: 38.4 | NA | 158016, patients with immunocompromised diseases | 158016, patients without chronic kidney diseases, chronic lung diseases, diabetes, and immunocompromised diseases | >14 | Full vaccination |  |
| **Cancer** | | | | | | | | | | | |
| Di Fusco (2021)  [2] | USA | RCS | BNT162b2 | Patients: NA, Control: 50 (17.5) | Patients: NA, Control: 39.7 | No | 70359, patients with cancer | 963962, patients without immunocompromised diseases | ≥14 | Full vaccination |  |
| Bellusci (2022)  [3] | USA | RCS | BNT162b2, mRNA-1273, Ad.26.COV2.S and combination | Patients: median (range) 70 (28-89), control 34.5 (21-75) | Patients: 56.3, Control: 31.3 | NA | 48, myeloid neoplasms | 16, health control | NA | Full and booster vaccination |  |
| La (2022) [4] | USA | RCS | BNT162b2, mRNA-1273 | Patients: median NA, control NA | Patients: median NA, control NA | No | 2470, multiple myeloma | 2470, patients without multiple myeloma | >14 | Full vaccination |  |
| Lee (2022) [5] | UK | CCS | ChAdOx1 nCov-19, BNT162b2, mRNA-1273 | Patients: median 71, control 59 | Patients: NA, control: NA | NA | 124998, cancer patients | 3929844, patients without cancer | NA | Booster vaccination |  |
| Mittelman (2022) [6] | Israel | RCS | mBNT162b2 | Patients: median (IQR) 70 (59-79), control 70 (59-78) | Patients: 52, Control: 52 | No | 32156, hematological neoplasms | 32156, patents without hematological neoplasms | 7-43 | Full vaccination |  |
| Raiker (2022) [7] | USA | RCS | NA | Patients: NA, Control: NA | Patients: NA, Control: NA | NA | 17384, skin cancer | 17384, patients without skin cancer | NA | Full and booster vaccination |  |
| Rooney (2022) [8] | USA | RCS | BNT162b2, mRNA-1273 | Patients: NA, Control: NA | Patients: NA, Control: NA | NA | 8515, cancer patients, including breast, endocrine, gastrointestinal, female reproductive, male reproductive, unspecified male/female, head and neck, leukemia, lymphoma, multiple myeloma, respiratory, skin, unspecified cancer | 902, patients without cancer | >14 | Full vaccination |  |
| Song (2022) [9] | USA | CCS | BNT162b2, mRNA-1273 | Patients: NA, Control: NA | Patients: NA, Control: NA | No | 64150, cancer patients, including breast, skin, gastrointestinal, prostate, female reproductive, endocrine, thoracic, leukemia, multiple myeloma, lymphoma, and other cancers. | 34515, patients without cancer | >14 | Full vaccination |  |
| Wang (a) (2021)  [10] | USA | RCS | BNT162b2, mRNA-1273, Ad.26.COV2.S | Patients: 68.0 (11.6), Control: 51.3 (20.8) | Patients: 51.5, Control: 44.4 | No | 1182, Multiple myeloma | 506106, patients without cancer | >14 | Full vaccination |  |
| Wang (b) (2022) [11] | USA | RCS | BNT162b2, mRNA-1273, Ad.26.COV2.S | Patients: 65.4 (15.8), Control: NA | Patients: 50.9, Control: NA | No | 5956, hematological malignancy including lymphoma, leukemia and multiple myeloid | 508457, patients without cancer | >14 | Full vaccination |  |
| Wang (c) (2022)  [12] | USA | RCS | BNT162b2, mRNA-1273, Ad.26.COV2.S | Patients: 68.7 (12.4), Control: 51.1 (20.9) | Patients: 46.5, Control: 44.9 | No | 45253, all cancer including bladder, breast, colorectal endometrial, hematologic, kidney, liver, lung, pancreatic, prostate, skin, and thyroid cancer | 508457, patients without cancer | >14 | Full vaccination |  |
| Gong (2022)  [13] | Canada | RCS | BNT162b2, mRNA-1273, Ad.26.COV2.S | Patients: 66.1 (14.0), Control: 66.0 (14.0) | Patients: 34.6, Control: 34.6 | NA | 289400, all cancer including solid and hematologic cancer | 1157600, patients without cancer | >14 | Full vaccination |  |
| **Immune-mediated inflammatory disorders** | | | | | | | | | | | |
| Ben-tov (2021) [14] | Israel | RCS | BNT162b2 | Patients: 47 (17), Control: 47 (17) | Patients: 50 Control: 50 | No | 12231, patients with inflammatory bowel disease | 36254, patients without inflammatory bowl | >14 | Full vaccination |  |
| Di Fusco (2021) [2] | USA | RCS | BNT162b2 | Patients: NA, Control: 50 (17.5) | Patients: NA, Control: 39.7 | No | 35475, patients with rheumatologic/other inflammatory condition | 963962, patients without immunocompromised diseases | ≥14 | Full vaccination |  |
| Singh (2022)  [15] | USA | CCS | BNT162b2, mRNA-1273, Ad.26.CoV2.S | Patients: 66 IQR (55-75), Control: 50 (33-65) | Patients: 48, Control: 43 | NA | 47303, patients with rheumatic disease | 536954, patients without rheumatic disease | ≥14 | Partial or full vaccination |  |
| Boekel (2022)  [16] | Netherlands | PCS | ChAdOx1 nCov-19, BNT162b2, mRNA-1273, Ad.26.CoV2.S, and combination | Patients: 53 (14), Control: 57 (13) | Patients: 37, Control: 33 | NA | 4192, patients with immune-mediated inflammatory disease | 822, health controls | ≥14 | Partial, full or booster vaccination |  |
| Furer (2022)  [17] | Israel | PCS | BNT162b2 | Patients: NA, Control: NA | Patients: NA, Control: NA | NA | 766, patients with autoimmune inflammatory rheumatic diseases | 140, patients with immunocompetent condition | NA | Full or booster vaccination |  |
| Hadjadj (2022)  [18] | France | PCS | BNT162b2 | Patients: 52 IQR (37.8-66.3), Control: NA | Patients: 25, Control: NA | No | 64, patients with systemic inflammatory diseases | 21, patients without systemic inflammatory diseases | >28 | Full vaccination |  |
| Lev-Tzion (2022)  [19] | Israel | RCS | BNT162b2 | Patients: 51 (16), Control: 51 (16) | Patients: 49, Control: 49 | No | 4946, patients with inflammatory bowel disease | 4946, patients without inflammatory bowel disease | NA | Full vaccination |  |
| Machado (2022)  [20] | UK | RCS | ChAdOx1 nCov-19, BNT162b2, mRNA-1273 | Patients: NA, Control: NA | Patients: NA, Control: NA | NA | 4604, patients with inflammatory/autoimmune rheumatic and musculoskeletal disease | 517, patients without inflammatory/autoimmune rheumatic and musculoskeletal disease | 0 or >14 | Partial and full vaccination |  |
| Sun (2022)  [21] | USA | RCS | ChAdOx1 nCov-19, BNT162b2, mRNA-1273, Ad.26.CoV2.S | Patients: 65 IQR (54-74), Control: 51 (33-66) | Patients: 26.1, Control: 42.8 | NA | 13445, patients with rheumatoid arthritis | Patients without immune dysfunction | >14 | Partial, full and booster vaccination |  |
| Stalman (2022)  [22] | Netherlands | RCS | ChAdOx1 nCov-19, BNT162b2, CX-024414, Ad.26.CoV2.S | Patients: NA, Control: NA | Patients: 37.7, Control: 32.8 | YES | 1593, patients with immune-mediated inflammatory diseases | 579, patients without immune-mediated inflammatory diseases | >14 | Full vaccination |  |
| **Organ transplant** | | | | | | | | | | | |
| Di Fusco (2021) [2] | USA | RCS | BNT162b2 | Patients: NA, Control: 50 (17.5) | Patients: NA, Control: 39.7 | No | 675, patients with solid organ transplant | 963962, patients without immunocompromised diseases | ≥14 | Full vaccination |  |
| Sun (2022)  [21] | USA | RCS | ChAdOx1 nCov-19, BNT162b2, mRNA-1273, Ad.26.CoV2.S | Patients: 59 IQR (47-67), Control: 51 (33-66) | Patients: 40.4, Control: 42.8 | Yes | 8688, patients with solid organ transplant | 629211, patients without immune dysfunction | >14 | Partial, full and booster vaccination |  |
| Vinson (2022) [23] | USA | RCS | BNT162b2, mRNA-1273, Ad.26.CoV2.S | Patients: NA, Control: NA | Patients: NA, Control: NA | No | 17352, patients with solid organ transplant | 1974246, patients without immunocompromised/immunosuppressed condition | >14 | Full or booster vaccination |  |
| Kulkarni (2022)  [24] | India | RCS | ChAdOx1 nCov-19, BBV152 | Patients: NA, Control: NA | Patients: NA, Control: NA | NA | 17, patients with liver transplant | 60, healthy control | >14 | Full vaccination |  |
| **HIV/AIDS** | | | | | | | | | | | |
| Di Fusco (2021)  [2] | USA | RCS | BNT162b2 | Patients: NA, Control: 50 (17.5) | Patients: NA, Control: 39.7 | No | 2103, patients with HIV/AIDS | 963962, patients without immunocompromised diseases | ≥14 | Full vaccination |  |
| Sun (2022) [21] | US | RCS | ChAdOx1 nCov-19, BNT162b2, mRNA-1273, Ad.26.CoV2.S | Patients: 51 IQR (37-60), Control: 51 (33-66) | Patients: 74.4, Control: 42.8 | NA | 8536, patients with HIV infection | 629211, patients without immune dysfunction | >14 | Partial, full and booster vaccination |  |
| Coburn (2022) [25] | USA | RCS | BNT162b2, mRNA-1273, Ad.26.CoV2.S | Patients: NA, Control: NA | Patients: 92.9, Control: 91.8 | Yes | 33029, patients with HIV infection | 80965, patients without HIV infection | >14 | Full or booster vaccination |  |

RCS: retrospective cohort study; PCS: prospective cohort study; CCS: case-control study; NA: not available.

Full vaccination was defined as two doses of an mRNA vaccine or Ad.26.COV2.S single dose.

Booster vaccination was defined as any vaccine dose after the second primary mRNA dose or Ad.26.COV2.S single dose.

**Table S3. Confounders adjustment and quality assessment of the included studies.**

| **Studies (years)** | **Adjusted parameters** | **Adjustment** |
| --- | --- | --- |
| Smits (2023) | NA | No |
| **Cancer** |  |  |
| Di Fusco (2021) | NA | No |
| Bellusci (2022) | NA | No |
| La (2022) | age and exact matching for other variables | Yes |
| Lee (2022) | age, sex, levels of deprivation, ethnicity, primary dose manufacturer and booster dose manufacturer | Yes |
| Mittelman (2022) | age bins of 5 years, sex, town of residence, number of influenza vaccinations during the preceding 5 years, pregnancy status, and bins of the total number of coexisting conditions | Yes |
| Raiker (2022) | comorbidities and demographics | Yes |
| Rooney (2022) | NA | No |
| Song (2022) | NA | No |
| Wang (a) (2021) | demographics, adverse socioeconomic determinants of health, transplant procedures, comorbidities, vaccine types, and medications | Yes |
| Wang (b) (2022) | NA | No |
| Wang (c) (2022) | demographics, adverse socioeconomic determinants of health, comorbidities, and vaccine types | Yes |
| Gong (2022) | aggregated clinical group score and receipt of third COVID-19 vaccination as time-varying covariate | Yes |
| **Immune-mediated inflammatory disorders** | | |
| Ben-tov (2021) | sex, birth year, coexisting comorbidities, and month of the first vaccination dose | Yes |
| Di Fusco (2021) | NA | No |
| Singh (2022) | age, gender, race/ethnicity, Quan-Charlson Comorbidity Index, and data partner | Yes |
| Boekel (2022) | NA | No |
| Furer (2022) | NA | No |
| Hadjadj (2022) | NA | No |
| Lev-Tzion (2022) | age, sex, jurisdiction of residence, health maintenance organization, and vaccination dates | Yes |
| Machado (2022) | NA | No |
| Sun (2022) | study period, full vaccination status, COVID-19 infection before, and immune dysfunction group | Yes |
| Stalman (2022) | NA | No |
| **Organ transplant** | | |
| Di Fusco (2021) | NA | No |
| Sun (2022) | Study period, full vaccination status, COVID-19 infection before, and immune dysfunction group | Yes |
| Vinson (2022) | NA | No |
| Kulkarni (2022) | NA | No |
| **HIV/AIDS** |  |  |
| Di Fusco (2021) | NA | No |
| Sun (2022) | Study period, full vaccination status, COVID-19 infection before, and immune dysfunction group | Yes |
| Coburn (2022) | Age, sex, race and ethnicity, primary vaccination series type, COVID-19 prior to full infection, 3-month calendar period, an interaction of COVID-19 prior to fully vaccinated and 3-month calendar period, and cohort | Yes |

NA: not available.

**Table S4. Newcastle-Ottawa Scale for risk of bias assessment of studies included in the studies.**

| **Author** | **Selection** | **Comparison** | **Exposure/Outcome** | **Total score** |
| --- | --- | --- | --- | --- |
| Smits (2023) | ☆☆☆ | ☆ | ☆☆☆ | 7 |
| **Cancer** | | | | |
| Di Fusco (2021) | ☆☆☆ | ☆ | ☆☆☆ | 7 |
| Bellusci (2022) | ☆☆☆ | ☆ | ☆☆ | 6 |
| La (2022) | ☆☆☆ | ☆☆ | ☆☆☆ | 8 |
| Lee (2022) | ☆☆ | ☆☆ | ☆☆ | 6 |
| Mittelman (2022) | ☆☆☆ | ☆☆ | ☆☆☆ | 8 |
| Raiker (2022) | ☆☆☆ | ☆☆ | ☆☆ | 7 |
| Rooney (2022) | ☆☆☆ | ☆ | ☆☆☆ | 7 |
| Song (2022) | ☆☆☆ | ☆ | ☆☆☆ | 7 |
| Wang (a) (2021) | ☆☆☆ | ☆☆ | ☆☆☆ | 8 |
| Wang (b) (2022) | ☆☆☆ | ☆ | ☆☆☆ | 7 |
| Wang (c) (2022) | ☆☆☆ | ☆☆ | ☆☆☆ | 8 |
| Gong (2022) | ☆☆☆ | ☆☆ | ☆☆☆ | 8 |
| **Immune-mediated inflammatory disorders** | | | | |
| Ben-tov (2021) | ☆☆☆ | ☆☆ | ☆☆☆ | 8 |
| Di Fusco (2021) | ☆☆☆ | ☆ | ☆☆☆ | 7 |
| Singh (2022) | ☆☆☆ | ☆☆ | ☆☆☆ | 8 |
| Boekel (2022) | ☆☆☆☆ | ☆ | ☆☆☆ | 8 |
| Furer (2022) | ☆☆☆☆ | ☆ | ☆☆ | 7 |
| Hadjadj (2022) | ☆☆☆☆ | ☆ | ☆☆☆ | 8 |
| Lev-Tzion (2022) | ☆☆☆ | ☆☆ | ☆☆ | 7 |
| Machado (2022) | ☆☆☆ | ☆ | ☆☆☆ | 7 |
| Sun (2022) | ☆☆☆ | ☆☆ | ☆☆☆ | 8 |
| Stalman (2022) | ☆☆☆ | ☆ | ☆☆☆ | 7 |
| **Organ transplant** | | | | |
| Di Fusco (2021) | ☆☆☆ | ☆ | ☆☆☆ | 7 |
| Sun (2022) | ☆☆☆ | ☆☆ | ☆☆☆ | 8 |
| Vinson (2022) | ☆☆☆ | ☆ | ☆☆☆ | 7 |
| Kulkarni (2022) | ☆☆☆ | ☆ | ☆☆☆ | 7 |
| **HIV** | | | | |
| Di Fusco (2021) | ☆☆☆ | ☆ | ☆☆☆ | 7 |
| Sun (2022) | ☆☆☆ | ☆☆ | ☆☆☆ | 8 |
| Coburn (2022) | ☆☆☆ | ☆☆ | ☆☆☆ | 7 |

**Table S5. Univariate meta-regression models of variables associated with the risk of breakthrough COVID-19 infections in vaccinated immunocompromised patients.**

| **Variables** | **Overall** | | **Cancers** | | **IMID** | |
| --- | --- | --- | --- | --- | --- | --- |
|  | **exp (b) (95% CI)** | **P** | **exp (b) (95% CI)** | **P** | **exp (b) (95% CI)** | **P** |
| Disease type | 1.08 (0.93-1.26) | 0.28 | - | - | - | - |
| COVID-19 history | 0.68 (0.49-0.94) | **0.02** | 0.62 (0.37-1.04) | 0.07 | 0.63 (0.41-0.96) | **0.04** |
| Study type | 1.09 (0.64-1.84) | 0.75 | 0.94 (0.42-2.10) | 0.87 | 1.04 (0.47-2.30) | 0.92 |
| Vaccine type | 1.42 (0.95-2.12) | 0.08 | 1.27 (0.58-2.75) | 0.50 | 1. 56 (1.02-2.37) | **0.04** |
| Country | 1.51 (1.08-2.10) | **0.02** | 1.39 (0.75-2.59) | 0.25 | 1.37 (0.83-2.29) | 0.19 |
| Breakthrough timing | 1.24 (0.78-1.98) | 0.35 | 1.11 (0.54-2.25) | 0.75 | 1.19 (0.52-2.73) | 0.64 |
| Vaccine dose | 1.19 (0.83-1.70) | 0.32 | 1.22 (0.56-2.66) | 0.58 | 1.13 (0.64-1.99) | 0.65 |
| Quality assessment | 1.48 (0.67-3.26) | 0.31 | 1.45 (0.59-3.58) | 0.37 | - | - |
| Adjustment | 0.84 (0.59-1.20) | 0.33 | 1.06 (0.52-2.17) | 0.86 | 0.88 (0.49-1.55) | 0.61 |

b: Coefficient; IMID: immune-mediated inflammatory disorders.

**Table S6. Subgroup analysis of the risk of breakthrough COVID-19 infections in vaccinated immunocompromised patients.**

| **Variables** | **Overall** | | | **Cancers** | | | **IMID** | | |
| --- | --- | --- | --- | --- | --- | --- | --- | --- | --- |
|  | **No. of studies** | **RR (95% CI)** | **P** | **No. of studies** | **RR (95% CI)** | **P** | **No. of studies** | **RR (95% CI)** | **P** |
| **COVID-19 history** |  |  | <0.01 |  |  | <0.01 |  |  | 0.36 |
| None | 12 | 1.82 (1.53-2.15) |  | 5 | 1.62 (1.36-1.93) |  | 4 | 1.54 (0.86-2.75) |  |
| Might enrolled | 16 | 1.28 (1.06-1.54) |  | 5 | 1.02 (0.96-1.08) |  | 6 | 1.17 (1.08-1.27) |  |
| **Study type** |  |  | 0.29 |  |  | 0.20 |  |  | 0.76 |
| CS | 25 | 1.51 (1.30-1.76) |  | 8 | 1.30 (1.13-1.49) |  | 9 | 1.29 (1.03-1.61) |  |
| CCS | 3 | 1.35 (1.19-1.54) |  | 2 | 1.43 (1.35-1.52) |  | 1 | 1.24 (1.18-1.31) |  |
| **Vaccine type** |  |  | 0.06 |  |  | **<0.01** |  |  | 0.31 |
| BNT162b12 | 9 | 1.97 (1.40-2.78) |  | 2 | 1.77 (1.45-2.16) |  | 5 | 1.53 (0.91-2.57) |  |
| Others | 19 | 1.38 (1.19-1.59) |  | 8 | 1.28 (1.10-1.47) |  | 5 | 1.17 (1.07-1.27) |  |
| **Country** |  |  | <0.01 |  |  | **<0.01** |  |  | **0.01** |
| USA | 17 | 1.67 (1.46-1.91) |  | 7 | 1.56 (1.31-1.85) |  | 3 | 1.47 (1.17-1.85) |  |
| others | 11 | 1.04 (0.98-1.10) |  | 3 | 1.04 (0.96-1.12) |  | 7 | 1.07 (0.95-1.20) |  |
| **Breakthrough timing** |  |  | 0.09 |  |  | 0.66 |  |  | 0.56 |
| >14 days | 21 | 1.53 (1.34-1.75) |  | 6 | 1.42 (1.21-1.68) |  | 7 | 1.28 (1.10-1.50) |  |
| Others | 7 | 1.20 (0.94-1.54) |  | 4 | 1.30 (0.89-1.89) |  | 3 | 1.08 (0.63-1.87) |  |
| **Vaccine dose** |  |  | 0.22 |  |  | 0.24 |  |  | 0.80 |
| Full vaccination | 16 | 1.61 (1.33-1.96) |  | 7 | 1.44 (1.23-1.68) |  | 5 | 1.33 (0.74-2.37) |  |
| Others | 12 | 1.37 (1.14-1.64) |  | 3 | 1.14 (0.79-1.63) |  | 5 | 1.23 (1.17-1.28) |  |
| **Adjustment** |  |  | **0.08** |  |  | 0.33 |  |  | 0.64 |
| Yes | 13 | 1.35 (1.20-1.52) |  | 6 | 1.27 (1.10-1.46) |  | 4 | 1.23 (1.17-1.29) |  |
| No | 15 | 1.61 (1.38-1.88) |  | 4 | 1.46 (1.14-1.88) |  | 6 | 1.36 (0.90-2.06) |  |

No.: number; RR: relative ratio; IMID: immune-mediated inflammatory disorders; CS: cohort study; CCS: case-control study.

**Table S7. Univariate meta-regression models of variables associated with the outcomes of breakthrough COVID-19 infections in vaccinated immunocompromised patients.docx**

| **variables** | **Univariate meta-regression** | |
| --- | --- | --- |
|  | **exp(b) (95% CI)** | **P** |
| COVID-19 history | 0.63 (0.24-1.62) | 0.30 |
| Study type | 1.16 (0.30-4.41) | 0.81 |
| Vaccine type | 0.86 (0.23-3.28) | 0.81 |
| Country | 0.96 (0.35-2.64) | 0.93 |
| Breakthrough timing | 1.41 (0.56-3.55) | 0.43 |
| Vaccine dose | 1.36 (0.50-3.70) | 0.51 |
| Adjustment | 0.57 (0.16-2.02) | 0.35 |

b: Coefficient.

**Table S8. Subgroup analysis of the outcomes of breakthrough COVID-19 infections in vaccinated immunocompromised patients.**

| **Variables** | **Immunocompromised patients** | | |  |
| --- | --- | --- | --- | --- |
|  | **No. of studies** | **RR (95% CI)** | **P** |  |
| **COVID-19 history** |  |  | 0.21 |  |
| None | 4 | 3.69 (2.15-6.35) |  |  |
| Might enrolled | 8 | 2.22 (1.24-3.97) |  |  |
| **Study type** |  |  | 0.77 |  |
| CS | 10 | 2.71 (1.79-4.12) |  |  |
| CCS | 2 | 2.43 (1.32-4.47) |  |  |
| **Vaccine type** |  |  | 0.77 |  |
| BNT162b12 | 2 | 2.43 (1.32-4.47) |  |  |
| Others | 10 | 2.71 (1.79-4.12) |  |  |
| **Country** |  |  | 0.97 |  |
| USA | 5 | 2.63 (1.38-5.00) |  |  |
| others | 7 | 2.67 (1.51-4.72) |  |  |
| **Breakthrough timing** |  |  | 0.37 |  |
| >14 days | 6 | 3.09 (1.75-5.45) |  |  |
| Others | 6 | 2.22 (1.42-3.50) |  |  |
| **Vaccine dose** |  |  | 0.44 |  |
| Full vaccination | 8 | 2.91 (1.76-4.80) |  |  |
| Others | 4 | 2.17 (1.25-3.78) |  |  |
| **Adjustment** |  |  | 0.73 |  |
| Yes | 9 | 2.47 (1.70-3.59) |  |  |
| No | 3 | 3.24 (0.74-14.26) |  |  |

CS: cohort study; CCS: case-control study.

**Reference**

1. Smits PD, Gratzl S, Simonov M, et al. Risk of COVID-19 breakthrough infection and hospitalization in individuals with comorbidities. *Vaccine.* 2023;41(15):2447-2455.

2. Di Fusco M, Moran MM, Cane A, et al. Evaluation of COVID-19 vaccine breakthrough infections among immunocompromised patients fully vaccinated with BNT162b2. *J Med Econ.* 2021;24(1):1248-1260.

3. Bellusci L, Grubbs G, Srivastava P, et al. Neutralization of SARS-CoV-2 Omicron after vaccination of patients with myelodysplastic syndromes or acute myeloid leukemia. *Blood.* 2022;139(18):2842-2846.

4. La J, Wu JT, Branch-Elliman W, et al. Increased COVID-19 breakthrough infection risk in patients with plasma cell disorders. *Blood.* 2022;140(7):782-785.

5. Lee LYW, Ionescu MC, Starkey T, et al. COVID-19: Third dose booster vaccine effectiveness against breakthrough coronavirus infection, hospitalisations and death in patients with cancer: A population-based study. *Eur J Cancer.* 2022;175:1-10.

6. Mittelman M, Magen O, Barda N, et al. Effectiveness of the BNT162b2mRNA COVID-19 vaccine in patients with hematological neoplasms in a nationwide mass vaccination setting. *Blood.* 2022;139(10):1439-1451.

7. Raiker R, Pakhchanian H, Deng M. 262 Safety and efficacy of the COVID-19 vaccine among fully vaccinated and boosted skin cancer patients. *J Invest Dermatol.* 2022;142(8):S44-S44.

8. Rooney A, Bivona C, Liu B, Streeter D, Gong H, Khan Q. Risk of SARS-CoV-2 Breakthrough Infection in Vaccinated Cancer Patients: A Retrospective Cohort Study. *J Hematol Oncol.* 2022;15(1):67.

9. Song Q, Bates B, Shao YR, et al. Risk and Outcome of Breakthrough COVID-19 Infections in Vaccinated Patients With Cancer: Real-World Evidence From the National COVID Cohort Collaborative. *J Clin Oncol.* 2022;40(13):1414-1427.

10. Wang L, Berger NA, Xu R. Risks of SARS-CoV-2 Breakthrough Infection and Hospitalization in Fully Vaccinated Patients With Multiple Myeloma. *JAMA Netw Open.* 2021;4(11):e2137575.

11. Wang L, Kaelber DC, Xu R, Berger NA. COVID-19 breakthrough infections, hospitalizations and mortality in fully vaccinated patients with hematologic malignancies: A clarion call for maintaining mitigation and ramping-up research. *Blood Rev.* 2022;54:100931.

12. Wang W, Kaelber DC, Xu R, Berger NA. Breakthrough SARS-CoV-2 Infections, Hospitalizations, and Mortality in Vaccinated Patients With Cancer in the US Between December 2020 and November 2021. *JAMA Oncol.* 2022;8(7):1027-1034.

13. Gong IY, Vijenthira A, Powis M, et al. Association of COVID-19 Vaccination With Breakthrough Infections and Complications in Patients With Cancer. *JAMA Oncol.* 2023;9(3):386-394.

14. Ben-Tov A, Banon T, Chodick G, et al. BNT162b2 Messenger RNA COVID-19 Vaccine Effectiveness in Patients With Inflammatory Bowel Disease: Preliminary Real-World Data During Mass Vaccination Campaign. *Gastroenterology.* 2021;161(5):1715-1717 e1711.

15. Mtei M, Mboya IB, Mgongo M, et al. Confidence in COVID-19 vaccine effectiveness and safety and its effect on vaccine uptake in Tanzania: A community-based cross-sectional study. *Hum Vaccin Immunother.* 2023;19(1):2191576.

16. Boekel L, Stalman EW, Wieske L, et al. Breakthrough SARS-CoV-2 infections with the delta (B.1.617.2) variant in vaccinated patients with immune-mediated inflammatory diseases using immunosuppressants: a substudy of two prospective cohort studies. *Lancet Rheumatol.* 2022;4(6):e417-e429.

17. Furer V, Eviatar T, Freund T, et al. Immunogenicity induced by two and three doses of the BNT162b2 mRNA vaccine in patients with autoimmune inflammatory rheumatic diseases and immunocompetent controls: a longitudinal multicentre study. *Ann Rheum Dis.* 2022;81(11):1594-1602.

18. Hadjadj J, Planas D, Ouedrani A, et al. Immunogenicity of BNT162b2 vaccine against the Alpha and Delta variants in immunocompromised patients with systemic inflammatory diseases. *Ann Rheum Dis.* 2022;81(5):720-728.

19. Lev-Tzion R, Focht G, Lujan R, et al. COVID-19 Vaccine Is Effective in Inflammatory Bowel Disease Patients and Is Not Associated With Disease Exacerbation. *Clin Gastroenterol Hepatol.* 2022;20(6):e1263-e1282.

20. Machado PM, Lawson-Tovey S, Strangfeld A, et al. Safety of vaccination against SARS-CoV-2 in people with rheumatic and musculoskeletal diseases: results from the EULAR Coronavirus Vaccine (COVAX) physician-reported registry. *Ann Rheum Dis.* 2022;81(5):695-709.

21. Sun J, Zheng Q, Madhira V, et al. Association Between Immune Dysfunction and COVID-19 Breakthrough Infection After SARS-CoV-2 Vaccination in the US. *JAMA Intern Med.* 2022;182(2):153-162.

22. Stalman EW, Wieske L, van Dam KPJ, et al. Breakthrough infections with the SARS-CoV-2 omicron (B.1.1.529) variant in patients with immune-mediated inflammatory diseases. *Ann Rheum Dis.* 2022;81(12):1757-1766.

23. Vinson AJ, Anzalone AJ, Sun J, et al. The risk and consequences of breakthrough SARS-CoV-2 infection in solid organ transplant recipients relative to non-immunosuppressed controls. *Am J Transplant.* 2022;22(10):2418-2432.

24. Kulkarni AV, Jaggaiahgari S, Iyengar S, et al. Poor immune response to coronavirus disease vaccines in decompensated cirrhosis patients and liver transplant recipients. *Vaccine.* 2022;40(48):6971-6978.

25. Coburn SB, Humes E, Lang R, et al. Analysis of Postvaccination Breakthrough COVID-19 Infections Among Adults With HIV in the United States. *JAMA Netw Open.* 2022;5(6):e2215934.
